# Microbiological profile in an ICU in 1 year

**DOI:** 10.1186/cc12965

**Published:** 2013-11-05

**Authors:** Joel Passos, Paulo Pires, Sérgio Curvelo, Decio Netto, Bruna Passos, Larissa Peixoto, Bruno D'Ávila

**Affiliations:** 1 Unimed Costa do Sol, Macaé, Brazil

## Background

Critical patients requiring prolonged intensive care are more at risk of being colonized by germs acquired in an ICU and present infections. The factors that contribute to the high rate of infection and mortality in ICUs are possibly associated with the severity of the underlying disease, invasive proceedings, the long period of hospitalization and use of antibiotics, especially the expanded spectrum, so that there are multidrug-resistant bacteria, which complicates therapy. Approximately 5% of patients admitted to ICUs will acquire a nosocomial infection, resulting in increased length of hospitalization, around 5 to 10 days, and will be considered a consequence of healthcare in 30% of cases. Diagnostic or therapeutic interventions provide breakdown of the mechanical barrier of the skin and mucus assigned to invasive, skin lesions caused by devitalization, trauma or by removing the skin secondary to burns or debridement. In addition to the mechanical factors that disrupt the natural barriers of defense, there are others that are inherent in clinical conditions of patients and promote the acquisition of infections in the hospital environment; the immune ability is compromised because the natural defense mechanisms are altered by the very nature base or as a result of therapeutic interventions. The rate of infection is high among intensive care patients, especially respiratory infections. *Pseudomonas aeruginosa *was the prevalent bacteria in our ICU. That is why the prevalence of infection acquired in the ICU is high and suggests that preventive measures are important to reduce the occurrence of infection in critical patients.

## Materials and methods

Retrospective study, analyzing culture results for 1 year in a ICU with 10 beds in the northern of state of Rio de Janeiro. We considered cultures of urine, blood, cerebrospinal fluid, tracheal aspirate, nasal swabs and catheter tip, and detected the most prevalent microorganisms in our ICU.

## Results

We analyzed 453 cultures, 178 (39.29%) were positive for some germ, 240 (52.98%) were negative and 35 (7.72%) had impaired analysis. Among the cultures were performed 152 blood cultures, 38 (25%) positive and 114 (75%) negative, 96 urine cultures, 36 (52.17%) positive and 60 (47.83) negative, 31 samples of tracheal secretions, 20 (64.51%) positive and 11 negative (35.49%), 141 nasal swabs, 71 (50.35%) positive and 70 negative (49.65%), and 27 cultures from the catheter tip, six (22.22%) positive and 21 (77.78%) negative. Among the positive blood cultures assayed as being prevalent was 31.57% with *P. aeruginosa*, the second *Staphylococcus aureus *and *Proteus mirabilis *with the same number of specimens, 15.78%. Among the 36 positive urine cultures, *Candida albicans *was the prevalent with 22.22%, second place was 13.88% *Escherichia coli *and *P. mirabilis *was third with 11.11%. The cultures were tracheal *P. aeruginosa *as the most prevalent in half of the cases (50%), and secondly *C. albicans *and *Acinetobacter baumannii *at 10%. Among the cultures of nasal swab taken on admission of patients, the prevalent germ was *P. aeruginosa *with 26.76%, in second place with 12.67% was *P. mirabilis *and third with the same number of cases were *A. baumannii *and *Serratia marcescens*, 11.26%. Among the catheter tip cultures, *P. aeruginosa *was prevalent with 40%, and *P. mirabilis *second with 20%. There no was positive cerebrospinal fluid culture in the period.

## Conclusions

This study contributes to the knowledge of local resistance rates, which is one of the basic steps for the establishment of individualized strategies regarding the use of antimicrobials.

